# Who defines good and bad? Boundaries of family decision-making in palliative care: reflections and reconstruction based on Eastern culture

**DOI:** 10.3389/fpsyg.2026.1924478

**Published:** 2026-08-21

**Authors:** Yanke Li, Man Teng Iong

**Affiliations:** 1 Faculty of Law, University of Macau, Taipa, Macao SAR, China; 2 Faculty of Law, Henan University of Urban Construction, Pingdingshan, Henan, China

**Keywords:** culture of filial piety, ethics and culture, family decision-making, palliative care, patient autonomy, patient welfare

## Abstract

In clinical practice in China, particularly in cancer treatment, family decision-making plays an important role. Over the past few years, efforts have been made across China to promote the implementation of palliative care. Influenced by traditional Chinese culture, an ethical conflict exists between patient autonomous decision-making and family-oriented choices driven by traditional filial piety. This study explores the ethical boundaries of family participation, focusing on how traditional filial piety and family interests affect the judgment of patients’ best interests within the Chinese cultural context. The analysis reveals that the Confucian virtue of filial piety may assist patients during treatment, but may infringe upon patients’ rights and complicate the process of informed consent. Through philosophical reflection, the study reconstructs a context-adapted ethical framework that prioritizes patient wellbeing, takes into account psychological dignity, and clarifies reasonable limits for family participation. The revised standard emphasizes informed consent, transparent communication, and culturally sensitive care, aiming to promote ethical, humane, and sustainable shared decision-making in oncology palliative care.

## Introduction

Throughout the long history of human practice, the definition of “good” and “bad” has always been a fundamental philosophical question (Arkhangelsky and Lei, 1983). This question becomes particularly complex in the field of palliative care at the end of life. When modern medicine possesses the technical capacity to prolong vital signs but cannot reverse the disease process or restore quality of life, the question “What is a good act?” is no longer a simple technical choice, but a profound issue of value judgment (Li, 2011). Is it “good” to use all medical means to maintain the survival of vital signs as far as possible, or is it “good” to follow the laws of nature, alleviate pain and maintain dignity to the end of life (Liao and Zhang, 2012; Zhang et al., 2018)? The answer to this question deeply shapes the fundamental conceptions of life, death, and morality held by patients, families, healthcare professionals, and society at large.

Within the mainstream discourse of Western biomedical ethics, the Four-Principles—autonomy, beneficence, non-maleficence, and justice—provides the foundational framework for medical decision-making (Zhu et al., 2020). Among these, the principle of respect for autonomy occupies a central position, emphasizing the patient’s right, as an independent individual, to make final decisions about their own body and life, provided they are fully informed (Selvamani, 2025). However, the universality of this highly individualistic ethical principle faces serious challenges when applied to diverse global cultural contexts.

Especially in the context of Eastern culture, the focus of ethical decision-making is completely placed on isolated individuals, which may conflict with the local profound cultural tradition and social structure (Wang, 2020). Therefore, the definition of “good” and “bad” in end-of-life decision-making is far from a simple universal formula, and it must be embedded in the context of specific cultural, social and family relations (Liu, 2012).

Different from the Western tradition of emphasizing individual rights, Chinese culture has always regarded individuals as a node within the network of relationships rather than an isolated atom. The family, as the most basic unit of society, carries the core functions of individual identity, emotional support, and attribution of responsibility (Chen, 2026). This cultural trait has profoundly affected medical practice, making family decision-making not only a common phenomenon in China, but also a widely accepted and expected cultural norm (Micollier, 2015). A 2022 study explicitly highlighted Confucian familism as a key framework for understanding shared decision-making at the end of life in the Chinese context (Yang et al., 2022).

Within this family-centered model of decision-making, when patients—particularly the elderly—face serious illness or reach the end of life, the power to make decisions often shifts to family members. At a superficial level, patients weakened by cancer may prevent from making rational decisions. On a deeper level, under the influence of Confucian relational ethics, patients voluntarily transfer decision-making authority to their family members.

Scholars have pointed out that this family-centered medical decision-making model can be traced back to Confucianism, which emphasizes the consensus reached through consultation within the family, rather than the absolute proposition of individual rights (Chen and Fan, 2010). Civil Code of the People’s Republic of China provides legal space for this model to a certain extent, stipulating in Article 1219 that when a patient is unable to express their will, close relatives have the right to make medical decisions (Civil Code of the People’s Republic of China, 2021). Therefore, in the practice of palliative care in China, it is impossible to explore ethical issues without the core element of family. Family members, especially the young ones, become the de facto ethical decision-makers for the old ones. They need to make life-and-death decisions on behalf of the patient in complex medical information, uncertain budget, heavy economic burden and deep emotions.

Although scholar has recognized the importance of Confucian culture and familism in Chinese medical decision-making (Yang et al., 2022), the existing research mostly stays in the phenomenon description. One scholar believes that the number of siblings in familism affects the choice of palliative care (Xu et al., 2019). There is no in-depth analysis of what specific reason in Confucian culture affects family decision-making. It is unknown whether it is good or bad to influence Chinese family decision-making based on what is judged.

In addition, the relevant laws, regulations and industry guidelines fail to provide clear boundaries regarding family decision-making authority. Although the state has issued documents such as the Practice Guide for Hospice Care, it also emphasizes the principle of fully informed and voluntary choice. However, these documents lack specific and clear provisions on the scope of family decision-making authority, especially how to deal with conflicts between patients’ wishes and family decision-making (Sun, 2024). The law gives close relatives the right to decide in certain circumstances, but does not provide ethical guidance on how to balance the best interests of patients, the past wishes of patients and the moral concepts of their families when exercising this right (Liu and Yang, 2022).

The purpose of this study is to fill this gap, and to systematically explore the ethical boundary of family decision-making in palliative care from the perspective of Confucian culture. This study will focus on answering the following core questions:

1. In the context of Chinese social culture, how does Confucian culture influence the definition of “good decision” and “bad decision” in palliative care?
2. What are the main ethical conflicts and dilemmas in this process?
3. How do we construct a decision support framework that respects Chinese traditional culture and conforms to the principles of modern bioethics?

This paper uses the method of philosophical analysis to discuss the influence of Chinese Confucian traditional culture on the decision-making of patients’ families. This study mainly analyzes how Confucian culture shapes the process of family joint decision-making, and discusses how to give reasonable restrictions on the participation of family joint decision-making under the principle of patient wellbeing, taking into account the patient’s psychological and personal dignity. This paper aims to promote more transparent communication and respect for cultural differences on the basis of emphasizing patients’ informed consent, so as to achieve more fair and humane medical decision-making.

## Methodology

To gain a deeper understanding of the Confucian culture in detail, this study selects the Confucian classic as the object of text analysis, including but not limited *The Analects of Confucius, Mencius, Zhongyong and The book of Rites* as the object of text analysis. To ensure accuracy and scholarly rigor in interpretation, the study relies on widely recognized annotated editions accepted within the academic community. All citations are presented in Harvard format, with clear source attribution, thereby maintaining transparency and methodological reliability. This textual analysis provides the philosophical foundation for examining how Confucian culture shapes family-centered decision-making in palliative care. The key conceptual terms and the research framework are set out in Table 1, which offers greater clarity on the study’s main content.

**Table 1:** The key conceptual terms and the research framework.

Category	Core concepts	Associated elements	Explanation in the paper
Rore question	Who define good and bad in family decision-making	What is a good act	In the context of Chinese social culture, how does Confucian culture influence the definition of “good decision” and “bad decision” in palliative care?
			What are the main ethical conflicts and dilemmas in this process?
			How do we construct a decision support framework that respects Chinese traditional culture and conforms to the principles of modern bioethics?
Decision mode	Western decision-making model	Respecting autonomy: centered on individual autonomy	Four principles: autonomy, beneficence, non-maleficence, justice
	Eastern decision-making model	Family-centered: joint decision-making	Family decision-making is influenced by the Confucian culture of filial piety. a. Filial piety is a form of gratitude toward parents. b. Filial piety is a family responsibility and emotional bond. c. Filial piety is a moral pressure.
Methodology	Hermeneutic circle	Part and whole	Provide a philosophical explanation of key elements within the context of Confucian culture.
Ethical Conflict	Autonomy and family involvement	Individual autonomy and Confucian familism	In traditional Confucian culture, individuals cannot be separated from family relationships, so individual’s wellbeing is linked to the family’s collective interests.
	Filial piety and patient wellbeing	Confucian gratitude and patient’s best interests	The family’s expectation to prolong life conflicts with the patient’s pursuit of quality of life.
	Disclosure and concealment	Informed consent and protective concealment	Family decision-making affects the patient’s right to informed consent.
	Cultural traditions and modern medical ethics	Family emotions and personal dignity	Excessive treatment affects the patient’s personal dignity and quality of life.
Proposed solution	Reconstructing the framework of family decision-making	Establishing a family decision-making model suitable for the Chinese cultural context	Prioritize patient wellbeing
			Protect the patient’s personal dignity
			Maintain transparency in communication

The text analysis of this study adopts the principle of Hermeneutic Circle proposed by Gadamer (Zhu, 2013). This means that the interpretation process is not linear, but repeated between the “part” and the “whole.” Specifically, the study seeks to grasp the overall position and significance of Confucian ethics within the whole philosophical system. The study deeply analyzes the specific sentences and paragraphs related to taking care of parents. Through a cyclical process involving the examination of specific textual content and an understanding of Confucian thought as a whole, the study ultimately achieves a profound level of understanding. Throughout this process, the researchers also consciously reflect on their own “preconceptions” (including their understanding of medical decision-making, autonomy and family relationships), striving to integrate these with Confucian classics in order to explore the cultural elements within the texts that influence family decision-making in medical diagnoses.

## Results

### Formation of the family-centered decision-making model: the influence of filial piety culture

Confucian thought regards filial piety (*xiao* in Chinese) as the foremost of all virtues, the cornerstone for maintaining family harmony and social order (Lin, 2025). Its meaning extends far beyond the material support of parents. It also covers respect, obedience, bringing honor to one’s ancestors, and the responsibility to do everything possible to secure medical treatment for one’s parents in the event of serious illness (Ma et al., 2025). This cultural gene has shaped China’s distinctive family-centered model of medical decision-making.

In sharp contrast to the Western model that emphasizes individual rights (including individual autonomy), the Chinese model believes that the family is a community of interests and emotions, in which an individual’s wellbeing is inextricably linked to the family’s collective interests (Liu, 2018). Within this framework, medical decisions are not merely personal choices but family affairs, deeply rooted in the ethical and cultural imperatives of filial piety.

In the context of palliative care, this means that when patients face end-of-life decisions, family members naturally participate deeply, often becoming the primary decision-makers. Such involvement is regarded not only as a right but also as an inescapable moral responsibility (Liu, 2018). Research indicates that many Chinese patients tend to entrust decision-making authority to their families, viewing this as an expression of trust and family cohesion (Xiao et al., 2022). Consequently, collective family deliberation and consensus are considered the pathway to achieving harmony, which represents a higher cultural value.

#### The dual nature of filial piety as an ethical driving force

In cancer care, decision-making often represents the most difficult moment for families. Filial piety, as an ethical driving force, presents a dual attribute. On the one hand, filial duty motivates people to assume responsibility and to care for their parents (Tu, 2017). On the other hand, filial duty also carries moral pressure, influencing their decision-making.

On the one hand, filial piety is the bond of responsibility and emotion (Guo and Hu, 2025).

Filial piety embodies the idea of repayment. As recorded in the Book of Rites: “*Rites are a form of repayment*” (*lizzhe, baoye*) (Zheng, 2020). Parents give life to their children and devote immense effort to raising them; this gratitude must be repaid throughout the child’s lifetime. When parents suffer from serious illness or face life-threatening conditions, seeking the best possible medical treatment to prolong their lives becomes the concrete expression of this repayment (Jiang et al., 2019). Family members—especially children—are willing to devote a great deal of time, energy, and money in caring for their ill parents (Chen and Wang, 2023). Filial piety is not only a responsibility but also an essential emotional bond that sustains relationships within the family (Hu, 2025). When the family faces a major crisis such as cancer, filial culture provides cohesion, offering a psychological support system for both patients and their relatives (Hu, 2025).

Within the cultural framework of filial piety, when families are faced with major decisions, they discuss matters together and reach a collective decision. Family members not only provide each other with emotional support, but also share the heavy burden of making such choices (Hu, 2025). The pressure of end-of-life decision-making is extraordinarily intense, and it is often difficult for any one individual to bear alone. Guided by filial culture, family members participate in collective deliberation and decision-making, thereby distributing the psychological pressure in the process of decision-making by collective discussion. Should the decision lead to an unfavorable outcome, the fact that it was a collective family decision can also, to some extent, alleviate the sense of guilt felt by the individual decision-maker (Xu et al., 2025).

On the other hand, filial piety also produces moral pressure (Tang, 2008). Filial culture serves as a moral standard for evaluating behavior. From the Confucian perspective, the family is a life community that shares honor and disgrace, where individual interests are closely bound to collective family interests. When parents fall ill, the entire family faces a crisis, and medical decision-making is naturally regarded as a family matter. In many cases, the family’s best interests are considered superior to the patient’s individual interests. If children choose palliative care, they may bear the stigma of being unfiliality within the family or society (Gu, 2015). Under such external moral pressure, children tend to opt for active treatment, seeking to demonstrate that they have fulfilled their filial responsibility and thereby safeguarded both parental dignity and family honor (Zhang, 2020).

#### Filial piety as loyalty and benevolence

In Confucian culture, Being unfilial is regarded as a lack of the moral virtues of ren (benevolence) and zhong (loyalty). (Guo and Hu, 2025). Filial piety is regarded as an essential component of loyalty, and it is primarily expressed through benevolence (ren) (Guo and Hu, 2025). This love extends from one’s relatives to others, and from those with whom one shares a blood relationship to strangers. Filial devotion to parents is the starting point of all forms of love (Ren, 2016; Xiao, 2025). Confucius stated: “*The benevolent person loves others*” (Zheng, 2020), meaning that a kind-hearted person shows care and respect for others. Mencius explained benevolence as the heart that cannot bear to see others suffer, indicating that every person possesses a natural sense of compassion and, when witnessing others in pain or illness, cannot help but offer care and support (Wang, 2019a,b).

In the context of cancer treatment, children likewise cannot bear to see their parents endure suffering, and thus seek more effective medical interventions (He, 2024). Furthermore, the benevolent heart cultivated within the family extends to affairs of the state. A person who fails to be filial to their parents cannot truly care for others; one who lacks benevolence cannot govern politically in ways that benefit the people (Yang and Zang, 2025). For this reason, family members may also choose active treatment as a way of establishing a positive social image.

Confucian filial piety is like two sides of a coin. It is not only the emotional cornerstone of connecting family members and providing warm care, but also the cultural root of imposing moral shackles and leading to irrational medical decisions. Understanding the duality of filial piety as an ethical driving force is paramount for analyzing the various ethical conflicts that arise in Chinese palliative care.

### Conflict between patient autonomy and family decision-making: the practical dilemma of filial piety

Under the dominance of Confucian filial piety and the family-centered decision-making model, palliative care practice reveals a series of profound ethical conflicts (Chen and Fan, 2010). At the core lies the tension between the patient’s individual autonomy, the authority of collective family decision-making, and the multiple interpretations of human dignity.

#### Family collective decision-making may infringe upon patient autonomy

When it comes to major decisions regarding cancer treatment, traditional Confucian culture in China tends to favor collective decision-making within the family unit, with the final decision being reached through internal family consultation. The patient’s wishes are often absorbed into collective will, and medical decisions shift from being individual choices to family-led collective decisions (Hu, 2025). By contrast, modern Western medical practice places respect for patient autonomy at its core (Wei et al., 2019). Modern medical ethics assume that the patient is an independent and rational rights-bearing subject, the guardian of their own best interests, capable of making personal choices based on an understanding of diagnostic and therapeutic information.

Yet defining “best interest” is not an easy task. Cancer patients may hope to improve their quality of life before death and to depart with dignity. From the perspective of filial piety, children should insist on effective treatment methods. In practice, this family-centered approach negates the patient’s own wishes. Some scholars have proposed the concept of relational autonomy to interpret the Chinese context (Gómez-Vírseda et al., 2019). Individual autonomy is formed and expressed within the network of family relationships, with patient decisions deeply influenced by family members’ feelings and the pursuit of family harmony (Wang et al., 2026). Patients may voluntarily set aside certain personal preferences and comply with family decisions in order to spare their children moral burdens and psychological pressure (Jiang et al., 2019). This represents a form of autonomy reconstructed within relationships, carrying a stronger collectivist character, though the boundary between such autonomy and the patient’s genuine personal will often remain blurred.

Benevolent concealment infringes upon the patient’s right to informed consent. From ancient times to the present, protective deception has been a common phenomenon in Chinese medical practice (Liu, 2021). Rooted in Confucian tradition, partial or complete concealment is often chosen on the grounds of protecting the patient’s psyche from overwhelming shock (Wang, 2019a,b). Family members regard such concealment as an expression of filial devotion and benevolence.

Article 1,219 of the Civil Code of the People’s Republic of China explicitly stipulates the physician’s duty of disclosure and the patient’s right to informed consent (Civil Code of the People’s Republic of China, 2021). Yet in Chinese clinical practice, physicians frequently request family members to sign the patient’s consent forms (Cui et al., 2024). Although the motivation is to shield the patient from the impact of bad news. When the patient’s right to informed consent is deprived, patients lose the opportunity to make life decisions in their final period of life. While the family’s choice may be driven by filial piety and love, such decisions do not necessarily align with the patient’s best interests and may even contradict the patient’s own wishes.

#### Family decision-making may violate the patient’s psychological and personal dignity

In Confucian culture, a child’s ability to prolong their parents’ lives is seen as an expression of filial piety. Based on this consideration, children often make collective decisions to pursue effective treatment, with the goal of extending their parents with cancer’s survival to live by even one more day. Yet such decisions may result in patients being connected to numerous tubes, suffering severe side effects, and enduring great pain (Wang, 2021). These treatments reduce the patient’s quality of life and make patients lose their personal dignity (Ma et al., 2020).

In end-of-life care, studies have shown that many family members, driven by filial ethics, believe that only by pursuing every possible treatment can they be considered dutiful toward their parents, or that sending their parents to live in high-cost intensive care unit (ICU) is the only way to demonstrate their filial devotion (Ma et al., 2020). Such excessive treatment, however, infringes upon patient dignity. Patients lose the opportunity to choose palliative care and find emotional relief. In fact, it is contrary to the Confucian culture’s pursuit of a good and dignified end to life.

## Discussion

### Construct the ethical framework of family decision-making boundary

In the face of complex ethical conflicts arising from filial piety, this study actively explores the development of an ethical framework and decision-making model adapted to the local cultural context, with the aim of balancing patient autonomy and family involvement to achieve truly “good decisions.”

#### The highest principle: safeguard the patient’s welfare and best interests

“Patient welfare first” is the core principle of medical ethics that has been upheld throughout history and across cultures. It requires healthcare professionals and all those involved in decision-making to prioritize the patient’s health, life and wellbeing above all other considerations (such as social and moral pressures, financial interests or the wishes of the family) when making any medical decision. Family joint decision-making must be based on the highest principle of “patient’s welfare first.”

Adhering to the principle of patient welfare first provides a value benchmark for medical decision-making. When there is a disagreement between patients and family members, “which choice is most suitable for the patient’s fundamental welfare” should be the ultimate standard of judgment and adjudication.

Adhering to the principle of patient welfare first can correct the practical deviations of filial culture. Traditional filial piety is sometimes rigidly understood as “obedience” or as “preserving life” at all costs, while neglecting the patient’s quality of life and dignity. By placing patient welfare at the center, the practice of filial piety can be redirected from mere formal compliance toward genuine concern for the parent’s true wellbeing. Authentic filial devotion means helping parents achieve the greatest possible comfort, peace of mind, and dignity while they are ill, rather than subjecting them to unnecessary suffering merely to satisfy the children’s moral conscience or social expectations.

#### A key principle: safeguard patients’ psychological wellbeing and personal dignity

Human dignity is the cornerstone of modern law and ethics. Article 38 of the Constitution of the People’s Republic of China explicitly stipulates that “*the personal dignity of citizens of the People’s Republic of China shall not be violated*” (Civil Code of the People’s Republic of China, 2021). In medical contexts, patient dignity is especially fragile, easily undermined both by the illness itself and by inappropriate medical practices. Within the framework of collective family decision-making, threats to patient dignity often appear under the guise of “love” and “protection.” For example, concealing the patient’s condition and depriving them of the right to know essentially treats them as incapable of bearing the truth or of taking responsibility for themselves—as if they were “persons without full capacity for action.” This constitutes a devaluation of their personal dignity.

Respecting patient dignity requires attention to three essential aspects:

Recognizing patient autonomy: When a patient is capable of making decisions, it must be acknowledged that they are the primary decision-maker. Family members are merely providers of information and supporters of the decision-making process, not as wielders of family authority.

Protecting informed consent: Patients have the right to know all truthful information about their bodies and illnesses. Any form of benevolent concealment must be subjected to extremely cautious evaluation and, wherever possible, carried out on the basis of the patient’s own expressed wishes. In most cases, families should be encouraged to face the truth together with the patient, with the medical team offering professional psychological support.

Respecting the patient’s personal values: Different individuals hold diverse views on life, suffering, and death. Medical decisions should not be based solely on clinical indicators; they must also take into account the patient’s personal values, life beliefs, and ultimate goals (Liu and Yang, 2022).

#### Upheld transparency in communication

Acknowledging the importance of the family does not mean granting family members unlimited decision-making authority. To safeguard the patient’s fundamental welfare and dignity, transparent communication must be upheld (Jiang et al., 2019), thereby establishing clear ethical and legal boundaries for family participation.

The exercise of family decision-making should adhere to established standards. When a patient possesses full decision-making capacity, the patient’s own will has the effect of not being negated.

When patients lose the decision-making ability and family members act as proxies, two considerations must guide the process: first, whether the patient has left advance medical directives, which should be strictly followed; second, if no advance directives exist, family members should refer to the patient’s past words, actions, as well as their values, making the final decisions based on what is in the patient’s best interests.

**Figure 1:**
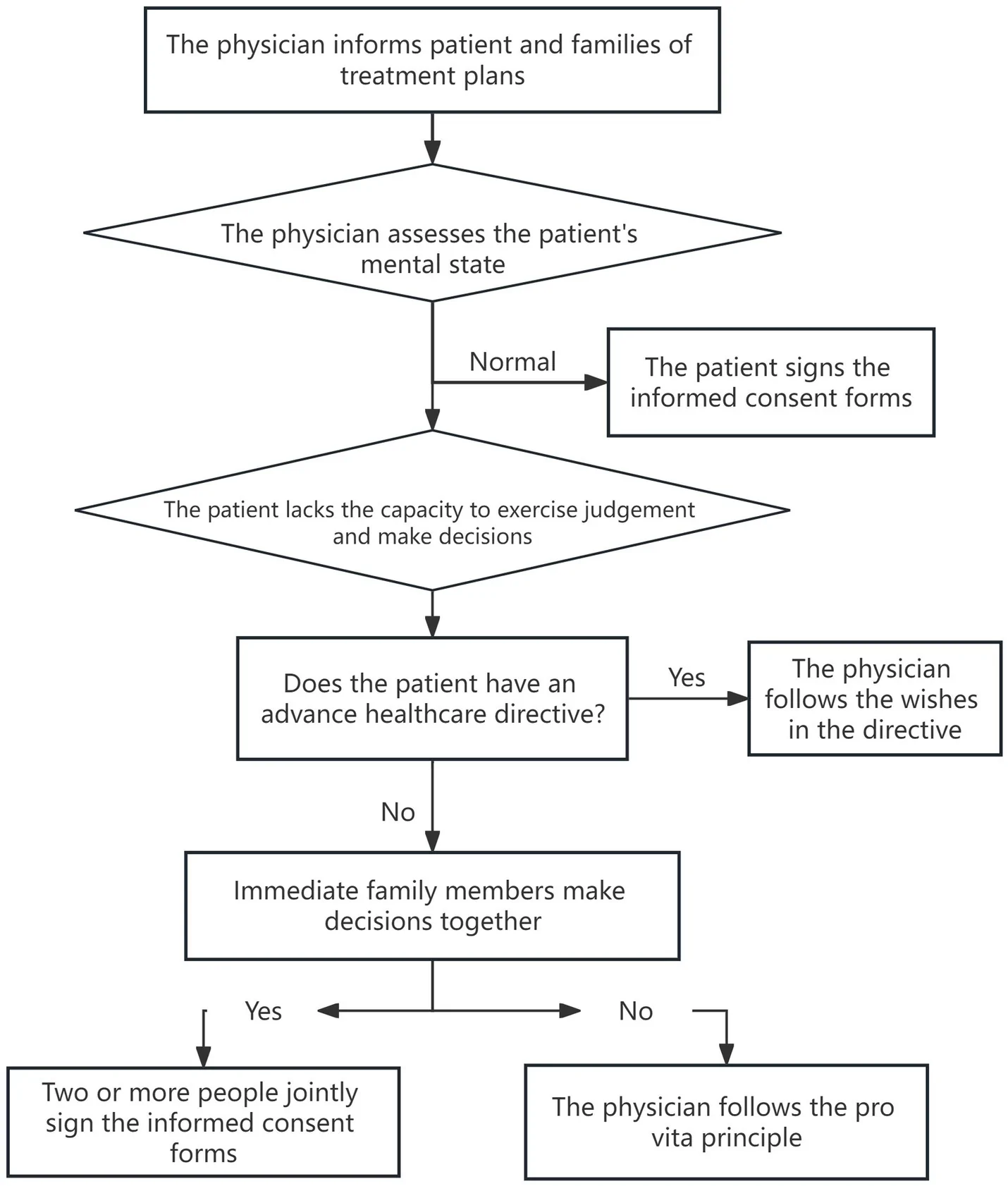
Informed consent signing process.

In practice, the decision-making process may be structured according to the model shown in Figure 1. When a patient’s decision conflicts with that of the family, the doctors must assess the patient’s mental capacity to determine whether they are capable of making an independent decision. If capable, priority is given to respecting the patient’s decision. If not, and when written advance directives exist, treatment must follow those directives. If there are no directives, the immediate family decides together, with two or more family members signing the informed consent form.

## Conclusion

This study has systematically examined how Confucian culture, as a deep-rooted foundation of Chinese society, profoundly shapes family decision-making patterns in clinical practice. This model demonstrates the unique ethical value of filial piety in fostering emotional support and responsibility within the family. At the same time, however, it generates conflicts with patient autonomy and individual dignity.

Through a comprehensive review and analysis of the literature, this paper has constructed a medical decision-making framework suited to China’s national context. The framework emphasizes the principle of patient welfare priority, insists on respecting patients’ psychological and personal dignity, and maintains appropriate boundaries for family involvement in decision-making.

This study also has two limitations. First, despite the adoption of a comprehensive search strategy, linguistic differences inevitably limited the inclusion of certain texts. Second, in modern Chinese society, family decision-making is influenced by multiple factors. This research focused solely on the cultural perspective of filial piety, without addressing other dimensions, such as household finances, living area, educational background. Future studies could employ empirical methods to analyze the roles of patients, family members, and medical staff in decision-making, thereby enriching our understanding of the diverse influences at play.
